# The effectiveness of ICT-supported flipped learning in an EFL context: A case of northern Iraq

**DOI:** 10.3389/fpsyg.2022.943956

**Published:** 2022-10-20

**Authors:** Abubakir Muhammad Mahmood, Behbood Mohammadzadeh

**Affiliations:** ELT Department, Faculty of Education, Cyprus International University, Nicosia, Turkey

**Keywords:** flipped learning, ICT, academic performance, students attitudes, teacher perceptions

## Abstract

Flipped Learning (FL) is a pedagogical model that leverages technology-enhanced instruction inside and outside of class time to maximize student engagement and learning during class time. This study investigated the effectiveness of ICT-supported Flipped learning in an EFL context in Northern Iraq to contribute to sustainable personalized language learning. The participants were EFL students and teachers of a primary school in Raniyah. The study employed an experimental method to collect student data and a teacher questionnaire. The results indicated that Flipped learning had statistically significant effects on the students’ academic performance and achievement. The mean scores of the post-tests scored higher than that of the pre-test. The mean scores of the EG with (*M* = 89.15) for the fifth grade and (*M* = 81.40) for the sixth grade were much higher than those of the CGs (*M* = 70.40) for the fifth grade and (*M* = 67.40) for the sixth grade which indicated that FL had a statistically great impact on the students’ academic performance and their product improvement. Besides, the results of the *t*-test showed a significant difference in performance between students in both EGs and CGs; the participants outstripped statistically significant on the post-test (*p* < 0.05) compared to the pre-test. The majority of the participants’ attitudes were positive toward Flipped learning, and they remarked that it had a significant role in learning compared to the conventional classes. The participant teachers quoted positive views on Flipped learning over traditional classes.

## Introduction and state of the question

Nowadays, ICT-supported education materials are widely being integrated into educational environments around the whole globe. This integration has provided educators with dynamic educational resources and has accelerated their capabilities in terms of both content and assessment (Ouda and Ahmed, 2016). Many countries are developing ways to embed in the educational system ICT-supported materials and facilities to enhance their educational outcome and achievements. The ICT-supported FL model provides a milieu in which interaction between student and teacher becomes a sustainable pattern (Tully, 2016). According to Bergmann and Sams (2012), in the FL model, understanding and remembering activities are fulfilled by students before class at home, and then activities related to applying, analyzing, evaluating, and creating are done in the classroom. Integrating the FL in the process of learning encourages students to bear the responsibility for the topic resulting in a more dynamic learning space, and the outcomes enhance self-efficacy among students (Namik et al., 2014). Providing students with various materials as before class activities enable students to participate actively in the classroom. Elian and Hamaidi (2018) highlight that the use of FL as a modern teaching method will empower students to use advanced techniques smartly and enjoyably, which meets the needs of students at present. The teacher’s role as a mediator and motivator accelerates the learning process both before and during the courses.

The current study aimed to show the effectiveness of FL in an EFL context at the primary stages in the North of Iraq. Since the process started changing the EL curriculum of the K-12 stage, teachers’ perspectives have changed following traditional methods toward communicative teaching approaches. Besides, the students were also more attracted to blended learning which enhances their academic performances. It was the first step in replacing the traditional method with blended learning (BL) toward the FL model. The researcher expected that the study’s results might be useful for future researchers interested in examining further effects of FL. On the other hand, the findings of this study might be significant for the basic school level who used ICT tools with the FL; meanwhile, there was very little study available to inform the decisions of basic educators who might be interested in implementing FL in their classrooms (Segolsson et al., 2017).

### Objectives and research questions

On the methodological range, FL is increasingly prevalent in learning and teaching processes, being used for different subjects and educational levels (Mengual-Andrés et al., 2020). Accordingly, the scientific literature brings together exploratory studies on the efficacy of this methodology in diverse contexts (Lin et al., 2019). However, little research has been found addressing flipped learning from an evaluative perspective of the skills for good staff teaching practices to materialize. Therefore, the objectives of this study are **(a)** to analyze and show the effectiveness of FL in an EFL context at the primary stages in the North of Iraq, **(b)** to show the Iraqi EFL learners’ perceptions of their learning experience with an FL approach vs. a traditional approach, and **(c)** to discover the educators’ perceptions of the student and parents and the instructional considerations in the FL in the North of Iraq.

Based on these objectives, the following research questions (RQ) were formulated and this study seeks to find answers to them:

• RQ_1_: Is there any statistically significant difference in performance levels between experimental and control group students?
• RQ_2_: What are the Iraqi EFL learners’ perceptions of their learning experience with an FL approach vs. a traditional approach?
• RQ_3_: How did the students perceive the platform selected for the online learning community?
• RQ_4:_ What are the educators’ perceptions of the student and parent considerations and the instructional considerations in the FL?

## Literature review

A literature review is one of the crucial elements of research studies. It gives an idea about already known affairs connected to the research area and also helps to interpret the findings as well as help to learn from previous mistakes (Bryman, 2015). In the FL model, direct learning is shifted out of the large group learning space and moved into the individual learning space by the teachers with the help of ICT devices. FL model is a kind of BL strategy that involves any employment of ICT devices to affect the learning process in the class. So instead of lecturing, the teacher can have more time to interact with students, thus providing more opportunities to deliver more personal feedback and assistance to pupils. Besides, they receive feedback from their peers about the activities they are performing and what they have not understood yet (Willey and Gardner, 2013). FL allows students and teachers to build interactive and communicative language learning in the classroom. It is so beneficial as an online practice to motivate learners in a collaborative way to engage and progress all four language skills as autonomous learning (Banditvilai, 2016). Several educators examined the influences of FL on EL skills, like listening (Ahmad, 2016), writing (Engin, 2014; Yu and Wang, 2016); grammar (Al-Harbi and Alshumaimeri, 2016). Yet, there is very little overview of the four skills of language under the impact of the FL model.

Many studies examined the FL effectiveness in developing ELT in various contexts all over the world (Basal, 2012; Pérez and Riveros, 2014; Huang and Hong, 2015; Hung, 2015; Bauer-Ramazany et al., 2016; Ekmekci, 2017; Farrah and Qawasmeh, 2018; Haghighi et al., 2018; Shotaro et al., 2018; Hazaymeh and Altakhaineh, 2019; Vaezi et al., 2019). Likewise, in this country, Qader and Arslan (2019) examined the effect of FLI on Iraqi EFL learners’ writing skills. The findings indicated that a statistically significant difference existed between the CG and EGs and more specifically, the students of the EG performed better on the writing tests than the students of the CG. The consequences showed the majority of the learners’ attitudes toward FLI were positive.

As it was specified by Educause (2012), the factual implementation of FL dates back to 2007, when two teachers of science in Woodland Park, Colorado, Sams and Bergmann, began helping their absent students with their prior recorded videos and screencasting to recoup for the lessons that their students missed because of different events. They posted the videos online to their classes during the 2007–2008 academic school year. The teachers asked the learners to take notes on the videos and come to class at least with one thoughtful question to ask and/or share. Then, they observed that students’ motivation and interest had been enhanced. Finally, the teachers together wrote a book titled “Flip your classroom: Reach every student in every class every day,” which rationalizes multiple other teachers and educators to successfully flip their classrooms (Bergmann and Sams, 2012). Since it was created in 2014, the Flipped Learning Network (FLN) has been a great official online platform for teachers and educators to master skills, share knowledge, and get resources before flipping their classes (Flipped Learning Network, 2019).

FL is relatively a new teaching method that highlights the active use of class time by converting the students’ and teachers’ traditional tasks outside and inside the classroom. Bergmann and Sams (2014) defined FL as “direct instruction delivered to the individual outside of class, and more strategic use of in-class time for group work and individualized attention” (p. xi). In FL, the role of the students as passive lecture listeners changes to active participants in the classroom activities (Davies et al., 2013; Baepler et al., 2014; O’Flaherty and Philips, 2015). The students are expected to study the notes that teachers provided them at home or watch pre-recorded lecture videos before attending class. They can review the videos at their own pace and pause to take notes and review the important points. The learners are expected to participate in the active process of learning in the classroom by studying in groups or individually (Roach, 2014). The teachers’ guide the individuals in the classroom and work as facilitators to help with troubleshooting and give them feedback when needed. Moreover, the learners have additional support and practice opportunities with the goal of the lesson. The Flipped Learning Network (FLN) leaders claimed that the classroom could be flipped by the teachers by sending the learners to read and watch educational videos at home but necessarily, it does not mean that the learning is being flipped. FLN stated that any flipped learning is based on the four pillars of F-L-I-P “flexible environment, learning culture, intentional content, and professional educator” (Flipped Learning Network, 2014).

## Methodology

This part of the research study presents an experimental method by which qualitative analyses are deployed in the study. The experimental method involves manipulating variables to determine if changes in one variable cause changes in another variable. The biggest advantage of the experimental method is its capability to disengage random factors since an experiment is mainly manipulated which promises accuracy in the study. The study also adopts a survey method to gain the perceptions of teachers. Henceforth, the methodology includes participants, data collection instruments, data collection procedures, and data analysis methods.

### Participants

This study used purposeful sampling techniques. Creswell (2008) confirmed that purposeful sampling is a method where “the inquirer selects individuals and sites for study because they can purposefully inform an understanding of the research problem and central phenomenon” (p. 125). Mainly, the participants of the study included the teachers and the pupils of (Bradost Private School) in the Raniyah district. They were 11 and 12-year-old EFL learners, studied English for 5–6 years and they are at A2 level according to CERF standards. The participants studied EFL during the Fall semester of the academic school year 2019–2020 with the FL model. The sample of the study was composed of four groups (fifth and sixth stages). The researcher divided them into two main groups [experimental Groups (EG) and Controlled Groups (CG)]. Two groups of the fifth stage, one group was EG, and the other was CG, and the other two groups were of the sixth stage, one group was EG, and the other group was a CG that they were assigned purposefully. While the EGs consisted of 40 students, 18 females and 22 males, the CGs were composed of 40 learners, 19 of whom were females and the rest 21 participants were males.

### Instruments

The researcher used various survey questionnaires as instruments to collect more suitable data from the participants to reach the answer to the research questions, (a) The Learning Experience Questionnaire (LEQ) consisted of 19 items and the statements were distributed over six concepts like *usefulness* (4) items, *autonomy* (4) items, *engagement* (4) items, *satisfaction* (3) items, *motivation* (3) items, and *anxiety* (1) item. (b) Technology Acceptance Model (TAM) consisting of 24 items that included five constructions *perceived ease of use* (5) items, *system characteristics* (6) items, *perceived usefulness* (5) items, *behavioral intention* (4) items, and *material characteristics* (4) items. And (c) Teachers’ Perceptions Questionnaire (TPQ) consisted of 17 items which included five constructions like: [*potential benefits of the pupils in FL* (3) items, *Instructional Considerations in the FL* (4) items, *Learning in the FL* (3) items, *Student Considerations in the FL* (5) items, *Parent Consideration in the FL* (2) items]. All the questionnaires had a 5-point Likert-type response format, that ranged from: Strongly Disagree (SD), Disagree (D), Neutral (N), Agree (A), and Strongly Agree (SA). The questionnaires aimed at exploring the language learners and the teachers’ perceptions of the availability of collaboration in the FL community and the accessibility and availability of online course materials, as well as their attitudes toward the FL approach. To analyze the collected data obtained through the above-mentioned instruments, the researcher also used the statistical software SPSS (version 25).

The other instruments used by the participants in the classroom to meet the instructional results goals of achieving performance in developing EL learning as ICT devices among participants were: students’ personal computers, laptops, iPods, tablets, smartphones, and EL syllabus textbooks for both stages were: “Sunrise for Kurdistan 5th and 6th” which designed for elementary EL learners who studied English for 5–6 years that they are in A2 level according to CERF standards. “The syllabus was almost a complete English course written, especially for primary and secondary school students. The course has a communicative approach, integrating *listening*, *speaking*, *reading*, and *writing* skills with a clear focus on grammar structures” (Amin, 2018, p. 138). Moreover, the researcher chose an online platform HAWRAFERGA (HRF), as a digital project web page for all of the KRG students in the North of Iraq (designed and adapted by a group of the Bradost school teachers) and another digital webpage OMED-DOST (OD) which was significantly specified for only the Bradost school program system to help the K-12 students with the entire subject syllabuses without teachers. OD has a “*question bank, text messaging, questioning, and answer”* between the individuals and teachers and the ability to post audio messages, files, and graphic “stickers” that express emotions or feelings collaboratively.

### Data collection procedures

This study relied on surveys, and it has become quantitative research because it provided numeric data from surveys. The researcher used the pre-test and post-test of both EG/CGs to assess the participant learners’ levels of listening comprehension, understanding, reading performance, and writing achievement before and after the treatment. Moreover, he used a survey questionnaire for the participant teachers to examine their deepest understanding of the situation and their attitude toward the FL model, in English which consisted of multiple choices. To help the pupil participants understand the statements completely, the survey questionnaires (LEQ and TAM) were used in the same format in both languages (English and Kurdish). After all, the responses were received back, they were analyzed and explained according to their categories. Then, they were compared with the previous research data discussed and ended with conclusions.

### Data analysis

As they were mentioned above, to answer the main research questions and state the aim of the study, the researcher used different instruments such as pre-test and post-tests for CG/EGs and three different questionnaires. Besides, the researcher used the statistical software SPSS (version 25) to analyze the collected data obtained through the above-mentioned instruments. In the beginning, two paired sample *t*-tests were run to analyze and provide an account of the improvement of the CG/EG from pre-test to post-test in the production of target learning activities for both stages. Then, to compare the post-tests of CG/EG an independent sample *t*-test was employed. As for the third research question, if there is a statically significant difference in performance levels between students in CG and EGs, a one-sample *t*-test was employed. To show the Iraqi EFL students’ perceptions about their learning experiences with FL instruction vs. the traditional approach, a descriptive statistical analysis was run to measure the average of their performance during the limited time in both CG and EGs. Furthermore, concerning the fourth research question, the attitudes of the participants toward the flipped learning experience and the selected online platform and two survey questionnaires were employed. Descriptive statistics were used to demonstrate the mean scores of the respondents in the flipped group for each item of the questionnaires. Quantitative analysis was used to explicate the effectiveness of FL in the process of education in this region.

## Findings

### Outcomes of students’ learning experience

**RQ_1_:** Is there any statistically significant difference in performance levels between experimental and control group students?

Table 1 shows the descriptive statistics comparing the pre-and post-tests in the CG, and the EGs presented that in both instructional modes, the mean scores of the post-test were higher than that of the pre-test. Comparing the post-test, the mean scores of the EG (*M* = 89.15) for the fifth grade and (*M* = 81.40) for the sixth grade were much higher than those of the CGs (*M* = 70.40) for the fifth grade and (*M* = 67.40) for sixth grade. Moreover, the paired-samples *t*-test shown in Table 2 suggested that the pupil participants outstripped statistically significant on the post-test (*p* < 0.05) compared to the pre-test in all four kinds of teaching models. To scrutinize whether there is a statistically significant difference between the mean scores of flipped and control groups in post-tests, an independent sample *t*-test was run.

**RQ_2_:** What are the Iraqi EFL learners’ perceptions of their learning experience with an FL approach vs. a traditional approach?

**TABLE 1:** Descriptive statistics of the pre-tests and the post-tests of the selected stages.

Paired samples statistics					
		N	Mean	Std. deviation	Std. error mean
Pre-test	Control group 5	20	45.70	13.49	3.01
	Experimental group 5	20	46.45	8.48	1.89
Post-test	Control group 5	20	70.40	15.05	3.36
	Experimental group 5	20	89.15	8.48	1.89
Pre-test	Control group 6	20	42.60	10.80	2.41
	Experimental group 6	20	44.00	6.79	1.51
Post-test	Control group 6	20	67.40	14.53	3.25
	Experimental group 6	20	81.40	14.25	3.18

**TABLE 2:** Paired *t*-test evaluation of the pre/post-test for the selected groups.

		Mean	SD	Std. error mean	Sig. (2-tailed)
CG5	Pre/post-test	24.70	7.30	1.63	0.000
EG5	Pre/post-test	42.70	11.10	2.48	0.000
CG6	Pre/post-test	24.80	7.72	1.72	0.000
EG6	Pre/post-test	37.40	11.86	2.65	0.000

The results indicated that the post-test scores of the EGs were significantly higher than those of the CGs (*p* < 0.05). to make more comparison between the CG and the EGs as is exposed in Table 2 Below, the results indicated that even in a couple of tests (Pre/Post-tests) scores, the EGs were significantly higher than those of the CGs (*p* < 0.05). Overall, the findings exposed that while both instructional models promoted the participants’ pragmatic competence, the FL contributed to better learning outcomes in both grades (fifth and sixth).

**RQ_3_:** How did the students perceive the platform selected for the online learning community?

To answer the *third* research question, the researcher used the Learning Experience Questionnaire (LEQ) to investigate the participants’ perception of the FL experience. The questionnaire statements characterized the concepts of usefulness, autonomy, engagement, satisfaction, motivation, and anxiety. The findings demonstrated in Table 3 specified the participants’ positive attitudes toward the FL, with the mean scores, respectively, of usefulness (*M* = 4.58), autonomy (*M* = 4.60), engagement (*M* = 4.64), satisfaction (*M* = 4.64), motivation (*M* = 4.69), and anxiety (*M* = 4.71). Accordingly, the FL improved the pupils’ pragmatic competence, made an autonomous learning environment for pupils, engaged the participant pupils in the activities of learning, made the pupils feel satisfied with their learning, and interested and motivated the pupils to learn the target speech act well, and helped the students to feel less anxious.

**TABLE 3:** Descriptive statistics of the perception of flipped classroom experience.

Constructs	Mean	Min	Max	Mode	N of the items
Usefulness	4.58	1	5	4	4
Autonomy	4.60	3	3	4	4
Engagement	4.64	3	5	5	4
Satisfaction	4.64	3	5	5	3
Motivation	4.69	1	5	4	3
Anxiety	4.71	4	5	5	1

The researcher employed TAM to explore the student respondents’ perceptions of using HRF as the online platform of the course and answer the *fourth* research question regarding the constructs realized in the questionnaire. Overall, the majority of the participants were in favor of using HRF for EL learning as it was provided with a detailed description of the results (Table 4). Among the presented results that TAM contained, perceived ease of use (*M* = 4.76) topped the rank, followed by system characteristics (*M* = 4.73), perceived usefulness (*M* = 4.72), behavioral intention (*M* = 4.70), and material characteristics (*M* = 4.69).

**RQ_4_:** What are the educators’ perceptions toward the student and parent considerations and the instructional considerations in the FL?

**TABLE 4:** Descriptive statistics of the Technology Acceptance Model (TAM).

Constructs	Mean	SD	Min	Max	N of the items
Perceived ease	4.76	0.503	1	5	5
System characteristics	4.73	0.540	3	5	6
Perceived usefulness	4.72	0.512	1	5	5
Behavioral intention	4.70	0.546	3	5	4
Material characteristics	4.69	0.562	3	5	4

To answer the *fourth* research question, the researcher used another questionnaire to explore the participant teachers’ perceptions about teaching and learning experience in the FLI through five constructs belonging to the questionnaire; (*potential benefits of the pupils in FL*, *Instructional Considerations in the FL*, *Learning in the FL*, *Student Considerations in the FL*, *Parent Consideration in the FL*). The researcher also used SPSS (version 25) and utilized descriptive and inferential statistics to analyze the collected data using means and standard deviations for each area investigated.

### Teachers’ perceptions of the potential benefits for students in the flipped learning

Table 5 shows a summary of the means and standard deviations of the several areas considered as potential benefits for students in the FL. The majority of the teachers agreed or strongly agreed in the area of the FL benefitting absent students (*M* = 4.62), struggling students (*M* = 4.62), and in-class and out-class time mean is (*M* = 4.00).

**TABLE 5:** Teachers’ perceptions of potential benefits for pupils in the FL.

No.	Area	Mean	Standard Deviation
S1	Absent students	4.62	0.51
S11	Struggling students	4.62	0.51
S6	In-class and out-of-class time	4.00	0.75

### Teachers’ perceptions associated with instructional considerations in the flipped learning

Table 6 presented the results of the means and standard deviations of the different areas associated with instructional considerations in the FL. Teacher participants agreed or strongly agreed in the areas of personalized learning (*M* = 4.50), pupils to teacher interaction (*M* = 4.37), active learning (*M* = 3.98), and time for learning (*M* = 4.25).

**TABLE 6:** Teachers’ perceptions associated with instructional considerations in the FL.

No.	Area	Mean	Standard Deviation
S10	Personalized learning	4.50	0.75
S16	Pupil-to-teacher interaction	4.37	0.74
S3	Active learning	4.37	0.74
S17	Time for learning	4.25	0.70

### Teachers’ perceptions associated with learning in the flipped learning

Table 7 provides the consequences of the means and standard deviations of the frequent areas regarding learning in the FL. In all three areas, the respondent teachers agreed or strongly agreed with passive learning (*M* = 4.50), EL learners (*M* = 4.37), and the pupils learning (*M* = 4.12) had a high level of agreement with the areas.

**TABLE 7:** Teachers’ perceptions associated with learning in the FL.

No.	Area	Mean	Standard deviation
S9	Passive learning	4.50	0.75
S5	EL learners	4.37	0.74
S12	Student learning	4.12	0.83

### Teachers’ perceptions associated with student considerations in the flipped learning

The results of Table 8 presented a summary of the means and standard deviations of the different areas considered with student considerations in the FL. Participant teachers agreed most strongly in the area of the student to student interaction (*M* = 4.37), classroom discipline (*M* = 4.12), accessibility to technology (*M* = 4.00), student responsibility (*M* = 3.87), and the area of student preference (*M* = 3.75) was the lowest mean.

**TABLE 8:** Teachers’ perceptions associated with student considerations in the FL.

No.	Area	Mean	Standard deviation
S15	Student-to-student interaction	4.37	0.74
S4	Classroom discipline	4.12	0.83
S2	Accessibility to technology	4.00	0.53
S14	Student responsibility	3.87	1.12
S13	Student preference	3.75	0.70

### Teachers’ perceptions associated with parent considerations in the flipped learning

Table 9 displays the results of the means and standard deviations of the varied areas considered with parent considerations in the FL. Teacher participants agreed or strongly agreed with the area of Parent or teacher conferences (*M* = 4.25), they believed that in a flipped classroom, video lectures make the class more transparent to parents, and also with the area of parent involvement (*M* = 4.25), they think that discussions with parent center were more on learning than they do on classroom behavior when using a flipped classroom.

**TABLE 9:** Teachers’ perceptions associated with parent considerations in the FL.

No.	Area	Mean	Standard deviation
S8	Parent or teacher conferences	4.37	0.51
S7	Parent involvement	4.25	0.88

## Discussion of the findings

This study aimed to explore the effectiveness of FL in an EFL context in the Northern part of Iraq. Through this article, the researcher focused on several instructional limitations as (a) whether the FL had a statistically great impact on the pupils’ academic performance and their product improvements, (b) to determine the Iraqi EFL learners’ perceptions due to their learning experience with FL approach vs. the traditional approach, (c) to explore the students’ perceives toward the platform selected for the online learning community, (d) to reveal the educators’ perceptions of the student and parent and the instructional considerations in the FL model.

To reach the aim of this study, the researcher analyzed the collected data, and the following results were revealed. Regarding the RQ_1_, the mean scores of the post-tests scored higher than that of the pre-test. The mean scores of the EG with (*M* = 89.15) for fifth grade and (*M* = 81.40) for the sixth grade were much higher than those of the CGs (*M* = 70.40) for fifth grade and (*M* = 67.40) for sixth grade, which indicated that FL had a statistically great impact on the pupils’ academic performance and their product improvement. Moreover, in answer to the question asked the learners, whether FL has any impact on the student participants’ performance and EL learning activities compared with regular classes, the majority of the respondents’ attitudes were positive and stated that FL had a significant role in the process of learning compared to the regular classes, and they had got great performance in EL learning during the amount of time they had practiced inside and outside of the class. In general, all the participants quoted a positive view of the FL over conventional classrooms. The findings of this study are in line with the results of Bergman and Sams (2013), Baranovic (2013), Mason et al. (2013), Schwanki (2013), and Hung (2015) in that flipped learning increased the students’ success and performance to a certain extent that confirmed the effectiveness of the FL in the process of education. Besides, the results of the *t*-test showed a significant difference in performance between participant students in both EGs and CGs; the pupil participants outstripped statistically significant on the post-test (*p* < 0.05) compared to the pre-test. The study’s results depicted an improvement in the participants’ pragmatic competence as well as their learning outcomes in both grades. The findings are in line with research conducted by Hung (2015, 2017), Amiryousefi (2017), Hsieh et al. (2017), and Lee and Wallace (2017) in which students in the flipped groups outperformed those in the non-flipped groups.

The researcher used LEQ to investigate the participants’ perception of the FL experience to answer the RQ_2_. The questionnaire statements characterized the concepts of *usefulness*, *autonomy*, *engagement*, *satisfaction*, *motivation*, and *anxiety*. The findings demonstrated and specified the participants’ positive attitudes toward the FL model with the mean scores, respectively, of *usefulness* (*M* = 4.58) which confirmed that the participants learned more and better in the FL than in regular classes, FL helped them to use EL more appropriately in real life, and at the result, FL had useful tools for increasing their knowledge and supporting their learning about the subject. With *autonomy* mean score (*M* = 4.60), it shows that through using FL, it became much more convenient to study at home by receiving immediate feedback. The *engagement* concept means score (*M* = 4.64) reveals that the participants engaged in spending more time and made more effort with having more opportunities to interact and practice than usual with FL. *Satisfaction* means a score (*M* = 4.64) tells us that they are deeply satisfied with the format structure and experience of the learning materials of the FL procedures. *Motivation* means score (*M* = 4.69) indicates that the student participants were eager to watch the videos and other materials set for this course before the class. Besides, the structure and format of FL motivated them to take the subject more seriously and spend more time with it. Finally, the *anxiety* means score (*M* = 4.71) confirmed that the use of the FL reduced the feeling of fear and tension because of the prior preparation for the class. These findings may also align with both Hung (2015) and Hsieh et al. (2017) who found FL pupils more engaged with course contents out of class in comparison with those in traditional classrooms.

To answer the RQ_3_ regarding the constructs comprehended in the questionnaire, the majority of the participants were in favor of using HRF for EL learning. The findings indicated that among the five constructs TAM contained, perceived ease of use mean score (*M* = 4.76) topped the rank, followed by system characteristics (*M* = 4.73), perceived usefulness (*M* = 4.72), behavioral intention (*M* = 4.70), and material characteristics (*M* = 4.69). Concerning *perceived ease of use*, they confirmed that using HRF to interact with instructors and peers was the covenant and gave them clear guidance about their school work. The findings for *system characteristics* revealed that HRF improved the participants’ ability of writing and proficiency in speaking based on the comments and suggestions made by their instructors and peers. Concerning *perceived usefulness*, the results revealed that using HRF to learn English was a brilliant idea, it strengthened the participants’ critical thinking. The findings regarding *behavioral intention* indicated that the majority of the respondents tolerate learning a language through HRF in the future. Finally, the result of the construct *material characteristics* specified that the audio/video lectures made and forwarded by the educators improved the participants’ oral proficiency and enhanced their pragmatic competence. These results displayed that HRF meets the criteria presented by Wu et al. (2012) for social media. They chose to make meaningful interactions outside the classroom to support simple implementation and self-direction for the sake of enabling the educator to engage and supervise pupils’ interactions. It is also in line with that of Hsieh et al. (2017), who used the LINE app as their online platform. Finally, the result of the construct material characteristics specified that the audio/video lectures made and forwarded by the educators improved the participants’ oral proficiency and enhanced their pragmatic competence.

To get more information and indicate further consequences about the effectiveness of FL in developing EL learning in an EFL context, the researcher used another questionnaire to explore the participant teachers’ perceptions about teaching and learning experience in FL through five constructs belonging to the questionnaire (potential benefits of the pupils in FL, Instructional Considerations in the FL, Learning in the FL, Student Considerations in the FL, Parent Consideration in the FL). The researcher also utilized descriptive and inferential statistics to analyze the collected data. Participant teachers strongly agreed in the area of student to student interaction (*M* = 4.37), classroom discipline (*M* = 4.12), accessibility to technology (*M* = 4.00), student responsibility (*M* = 3.87), and the area of student preference (*M* = 3.75) was the lowest mean. The participant teachers believed that it is challenging for many students to access those materials because of the lack of ICT facilities. This outcome is in line with other studies that stated the due to lack of technical requirements as being a potential barrier for students in the FL (Bergmann et al., 2012; Butrymowicz, 2012; Fulton, 2012; Milman, 2012). Besides decreasing pupils’ discipline issues, FL allowed them to develop better relationships with their peers through cooperation and collaboration. Furthermore, they have a sense of responsibility for their learning in FL, and they prefer FL over the traditional one. Teacher participants agreed or strongly agreed with the area of Parent or teacher conferences (*M* = 4.25), they believed that FL video lectures make the class more transparent to parents, and also with the area of parent involvement (*M* = 4.25), they think that discussions with parent center were more on learning than they do on classroom behavior when using an FL. Consequently, depending on the following construct mean scores, with instructional considerations in the FL, in the areas of personalized learning (*M* = 4.50), pupils to teacher interaction (*M* = 4.37), active learning (*M* = 3.98), and time for learning (*M* = 4.25), teacher participants were strongly agreed that time created for in-class activities in the FL allowed for more active learning and increased higher-order thinking for pupils. They also think that FL allowed teachers more time to personalize and increase instruction for pupils. These findings are in line with (Knight and Wood, 2005; Freeman et al., 2007) who have pointed out the benefits of utilizing active learning in the classroom and that active learning can lead students to higher-order thinking (Wette, 2011). Many recent studies highlight that using the FL contributes to the process of active learning in the classroom (Lage and Platt, 2000; Kaner and Fiedler, 2005; Day and Foley, 2006; Drumheller and Lawler, 2011; Valenza, 2012; Herreid and Schiller, 2013). In general, FL created time for direct instruction, active learning activities, and content coverage.

## Implications and recommendations

This article did not only provide a set of theoretical implications and recommendations, but it also at a practical level encouraged to elaborate contribution of varied mediators who achieved a part in the educational process like legislators, councilors, advisors, and researchers as well the educators or the teaching staff themselves. The crucial consideration lies in the integration of ICT and/or online platforms as an instructional method taking advantage of various potentials that might be produced of new inputs for the creation and consolidation of new involvements.

Besides, it is very important to help the educators with compulsory training so that they can make ICT and/or the new educational technology the premium teaching support within their career. This will inspire the expansion of the mandatory instructive and training activities which will contribute to improving didactic processes. Accordingly, this research also helps other educational establishments to develop training processes by this knowledge and information society. Furthermore, the conclusions of this paper study might be cooperative for elementary school educators’ training to use FL in an operative and effective technique.

The FL model is notably a new and effective method in the field of education in this region. Thus, educators, policymakers and education process stakeholders will follow and pay great attention to the significance of this article study. Consequently, the obtained information from the study will significantly have a great impact on the teaching and learning process by enabling the elementary school educators, policymakers, and school stakeholders at all stages to develop performances that simplify the implementation of FL in the classroom, and to take benefit from FL strategy as an operative technique to increase language learners’ engagement inside and outside the classroom and increase academic and personal skills for language learners.

Based on reviewing the literature, revealed that there is no current academic study addressing the role of professional development to provide support for allowing school teachers and stakeholders to use and apply FL in their classrooms or enabling those scholars who have rich experiences in applying FL in the classroom and/or to train school teachers to use it successfully. Through this research article, the researcher suggests for future research target the effectiveness of FL as a facilitative professional development at the district level.

Moreover, the other future researchers might comprise a larger sample size that would tolerate a mixed-method and/or quantitative or qualitative approach that might be connected to this study’s key findings. For example, the other future investigators might conduct similar data instruments, but with a greater sample size (several schools and their students) that would contribute a broader range of responses and would be more generalizable to the larger population.

In addition, the other future research could include the effectiveness of the FC/FL model in developing the English language skills together and/or separately alone as listening comprehension skills, reading comprehension skills, writing skills, and/or speaking skills) in an EFL context in this district and/or in the KRG (the Northern Part of Iraq), to examine the role and the impact of the FL strategy in the learning and teaching process over the language learners, instructors, parents, stakeholders, policymakers, and the districts as well.

The consequences of the involvement of Bradost school educators in this paper study who employed the FL model in their classrooms are not ideal; but their knowledge and familiarities shed light on the field areas that are in need and interest to be paid more attention for professional development supports, training to facilitate additional positive outcomes, parent support, and additional practice for using FL model in the classroom. Basic and preparatory school educators might need to deliberate how their existing knowledge might be enhanced with the upcoming implementation of professional development and support from parents. Another valuable attention is making a balance between the restricted ICT tools as instructional technology resources and the subject materials to have a change in the instruction model and technique from traditional to FL for the sake of proving more advantageous to educators, language learners, and parents as well.

Additionally, basic school educators who adopt and decide to use and apply the FL method in their classroom have to be aware of its utility, its practices, its effectiveness, its challenges and its instructional technology materials. Having an idea of what to expect from the educators might support both the educators and language learners partake in a positive and effective experience with the FL model in their classrooms. With the plans and techniques mentioned in this paper study, the basic school educators used to help their language learners highlight and deal with the new method of teaching and learning which inform the parents to select new ICT tools for the FL model in the classroom. Parents can also make an informed choice among the schooling options based on their student’s educational needs and aspirations.

The implications from this paper study for FL in the basic schools extend to backing for the language learners’ parents for high-quality teaching and training through technology devices. In this regard, parents and even educational stakeholders might not be alert of the expediency and positiveness of FL in basic schools and its constructive effect on language learners’ active performance in the classroom.

The use of FL at the basic and even at the preparatory school levels and the supplementary access to the internet service provided by schools have the opportunity to lessen the effects of low socioeconomic status on the children’s cognitive development as language learners. By guaranteeing that children have opportunities for having classes with the FL model early in their progress, they have the opportunity to improve English language skills (listening comprehension, speaking, reading comprehension, and writing skills) as they need to effect and control ICT tools for learning throughout their pre-recorded and live videos.

Additionally, the outcomes in this paper study specify the need for the districts to support fundamental and preparatory school educators to implement the FL strategy and method in their classrooms throughout educational training programs and workshops. It is also essential for school educators to be provided with ICT tools as the instructional technology resources and materials which progress the FL model in the classroom and foster student learning.

As professional development, workshop and/or training that emphasize the best critical instructional methods to support techniques and strategies for succession of the language learners should be provided to classroom educators. Professional development would also emphasize training and preparing the school educators and professional developers to efficiently deal with novel methods and techniques of education, such as the FL model which should be arranged using ICT tools as well, for the sake of improving school issues., increasing school capacity in these ways would progress the overall learning conditions for language learners. In this regard, Flipped educators have recommended switching from a teacher-centered classroom to a student-centered classroom and emphasize that switching from “sage on the stage” to “guide on the side” is a crucial element of flipped learning.

## Conclusion

In the conclusion, the findings showed that the participant learners performed notably better in the post-test of the EGs than they did in the pre-test of the Class Test. The EGs pupils’ attitudes toward FL were positive, and they confirmed FL had statistically a great impact on their academic performance and productivity improvement compared to the regular classes. The findings also exposed that while both instructional techniques promoted the participants’ pragmatic competence, the FL contributed to better learning outcomes in both grades (fifth and sixth). The participants also more convenient to study at home by receiving immediate feedback and getting enough time to practice and develop their self-study skills. Overall, FL improved the pupils’ pragmatic competence, made an autonomous learning environment for pupils, made them feel satisfied with their learning, interested and motivated to learn the target speech act as well, and helped the students to feel less anxious. This study also confirmed that using HRF and the ICT tools was a brilliant idea to learn English that strengthened critical thinking, improved students’ skills, and enhanced their production in class.

Among the limitations of this study was the fact of having applied a non-probabilistic study sample method, which was why the results obtained here should be used with caution, and above all, if the intention is generalized to other contexts. Besides, the limitation of the internet access to view videos and other FL resources was not always available and good in quality for all the students which sometimes affected the children’s cognitive development for their low socioeconomic status as language learners. In addition, to be able to access the sample, this study recommended that educators must carefully consider the accessibility to the FL regarding ICT tools to inspire and enhance the process of education. The goal of coming research would be to analyze the training of teaching staff at other educational stages, such as Higher Education and/or Primary Education, regarding the development and application of the FL method in the instructional processes. Likewise, including a qualitative analysis approach in future studies to complement the findings presented here would be interesting.

## Data availability statement

The original contributions presented in this study are included in the article/supplementary material, further inquiries can be directed to the corresponding author.

## Ethics statement

Ethical review and approval was not required for the study on human participants in accordance with the local legislation and institutional requirements. Written informed consent to participate in this study was provided by the participants’ legal guardian/next of kin.

## Author contributions

AM contributed to the conception and design of the study, organized the database, performed the statistical analysis, wrote the first draft of the manuscript, and wrote sections of the manuscript. BM contributed to the manuscript revision and approved the submitted version. Both authors contributed to the article and approved the submitted version.

## References

[B1] Ahmad S. Z. (2016). The flipped classroom model to develop Egyptian students’ listening comprehension. *English Lang. Teach.* 9 166–178. 10.5539/elt.v9n9p166

[B2] Al-Harbi S. S. Alshumaimeri Y. A. (2016). The flipped classroom impact in grammar class on efl saudi secondary school Students’ performances and attitudes. *Engl. Lang. Teach*. 9, 60–80.

[B3] Amin M. Y. M. (2018). The effectiveness of “Training course for English teachers in Iraqi Kurdistan” and improving teachers’ confidence. *Int. J. Human. Cultural Stud.* 5 137–148.

[B4] Amiryousefi M. (2017). The incorporation of flipped learning into conventional classes to enhance EFL learners’ L2 speaking, L2 listening, and engagement. *Innov. Lang. Learn. Teach.* 13 147–161. 10.1080/17501229.2017.1394307

[B5] Baepler P. Walker J. D. Driessen M. (2014). It’s not about seat time: blending, flipping, and efficiency in active learning classrooms. *Comp. Educ.* 78 227–236. 10.1016/j.compedu.2014.06.006

[B6] Banditvilai C. (2016). Enhancing students’ language skills through blended learning. *Electronic J. e-Learn.* 14 220–229.

[B7] Baranovic K. (2013). *Flipping The First-Year Composition Classroom: Slouching Toward The Pedagogically Hip.* Master’s dissertation, Missouri, DE: School of Graduate Studies of Southeast Missouri State University.

[B8] Basal A. (2012). “The use of flipped classroom in foreign language teaching,” in *Proceedings of the 3rd Black Sea ELT Conference Proceedings*, (Turkey).

[B9] Bauer-Ramazany C. Grany J. M. Marshall H. Sahieb C. (2016). Flipped learning in TESOL: definitions, approaches, and implication. *TESOL J.* 7 429–437. 10.1002/tesj.250

[B10] Bergman J. Sams A. (2012). *Flip Your Classroom: Reach Every Student in Every Class Every Day, USA: Iste.* Alexandria, VA: ASCD.

[B11] Bergmann J. Overmyer J. Willie B. (2012). *The Flipped Class: Myths Versus Reality.* Toronto, ON: The Daily Riff.

[B12] Bergmann J. Sams A. (2012). *Flip your Classroom: Reach Every Student in Every Class Every Day.* Washington, DC: International Society for Technology in Education.

[B13] Bergmann J. Sams A. (2014). *Flipped Learning: Gateway to Student Engagement.* Washington, DC: International Society for Technology in Education.

[B14] Bryman A. (2015). *Social Research Methods.* Oxford: Oxford university press.

[B15] Butrymowicz S. (2012). *Promise of the flipped classroom eludes poorer school districts. Hechinger report*. Available online at: http://hechingerreport.org/promise-of-the-flipped-classroom-eludes-poorer-school-districts/

[B16] Creswell J. W. (2008). *Educational Research: Planning, Conducting, and Evaluating Quantitative and Qualitative Research.* Upper Saddle River, NJ: Pearson/Merrill Prentice Hall.

[B17] Davies R. S. Dean D. L. Ball N. (2013). Flipping the classroom and instructional technology integration in a college-level information systems spreadsheet course. *Educ. Tech Res.* 61 563–580. 10.1007/s11423-013-9305-6

[B18] Day J. A. Foley J. D. (2006). Evaluating a web lecture intervention in a human-computer interaction course. *IEEE Trans. Educ.* 49 420–431. 10.1109/TE.2006.879792

[B19] Drumheller K. Lawler G. (2011). Capture their attention: capturing lessons using screen capture software. *College Teach.* 59:93. 10.1080/87567550903252793

[B20] Educause (2012). *7 Things you should know about Flipped Classrooms.* Denver, CO: Educause.

[B21] Ekmekci E. (2017). The flipped writing classroom in Turkish EFL context: a comparative study on a new model. *Turkish Online J. Distance Education-TOJDE* 18 151–167. 10.17718/tojde.306566

[B22] Elian S. M. Hamaidi D. A. H. (2018). The effect of using flipped classroom strategy on the academic achievement of fourth grade students in jordan. *Int. J. Emerg. Technol. Learn. (iJET)* 13 110–125. 10.3991/jet.v13i02.7816

[B23] Engin M. (2014). Extending the flipped classroom model: developing second language writing skills through student-centred digital videos. *J. Scholarship Teach. Learn.* 14 12–26. 10.14434/josotlv14i5.12829

[B24] Farrah M. Qawasmeh A. (2018). English students’ attitudes towards using flipped classrooms in language learning at hebron university. *RELP* 6 275–294.

[B25] Flipped Learning Network (2019). *The Four Pillars of F-L-I-P.* Available online at: http://fln.schxoolwires.net//site/Default.aspx?PageID=92 (accessed September 30, 2021).

[B26] Flipped Learning Network (2014). *Flipped learning*. Available online at: http://flippedlearning.org/cms/lib07/VA01923112/Centricity/Domain/46/FLIP_handout_FNL_Web.pdf (accessed August 11, 2019).

[B27] Freeman S. O’Connor E. Parks J. W. Cunningham D. H. Haak D. D. Wenderoth M. P. (2007). Prescribed active learning increases performance in introductory biology. *CBE Life Sci. Educ.* 6 132–139. 10.1187/cbe.06-09-0194 17548875 PMC1885904

[B28] Fulton K. P. (2012). 10 reasons to flip. *Phi Delta Kappan* 94 20–24. 10.1177/003172171209400205

[B29] Haghighi H. Jafarigohar M. Khoshsima H. Vahdany F. (2018). Impact of the flipped classroom on EFL learners’ appropriate use of refusal: achievement, participation, perception. *Comp. Assisted Lang. Learn.* 32, 261–293. 10.1080/09588221.2018.1504083

[B30] Hazaymeh W. A. Altakhaineh A. R. (2019). The effect of flipped classroom instruction on developing emirati EFL learners’ pragmatic competence. *Int. J. Learn. Teach. Educ. Res.* 18 89–111.

[B31] Herreid C. F. Schiller N. A. (2013). Case studies and the flipped classroom. *J. College Sci. Teach.* 42 62–66.

[B32] Hsieh J. S. Wu W.-C. V. Marek M. W. (2017). Using the flipped classroom to enhance EFL learning. *Comp. Assisted Lang. Learn.* 30 1–21. 10.1080/09588221.2015.1111910

[B33] Huang Y. N. Hong Z. R. (2015). The effects of a flipped English classroom intervention on students’ information and communication technology and English reading comprehension. *Educ. Technol. Res. Dev.* 64 175–193. 10.1007/s11423-015-9412-7

[B34] Hung H.-T. (2015). Flipping the classroom for English language learners to foster active learning. *Comp. Assisted Lang. Learn.* 28 81–96. 10.1080/09588221.2014.967701

[B35] Hung H. T. (2017). Design-based research: redesign of an English language course using a flipped-classroom approach. *TESOL Quarterly* 51 180–192. 10.1002/tesq.328

[B36] Kaner C. Fiedler R. L. (2005). “Inside out: a computer science course gets a makeover,” in *Paper Presented at the Twenty-eighth Convention of the Association for Educational Communications and Technology*, (Orlando, FL).

[B37] Knight J. K. Wood W. B. (2005). Teaching more by lecturing less. *Cell Biol. Educ.* 4, 298–310. 10.1187/05-06-0082 16341257 PMC1305892

[B38] Lage M. J. Platt G. J. (2000). The Internet and the inverted classroom. *J. Econ. Educ.* 31:11. 10.2307/1183335

[B39] Lee G. Wallace A. (2017). Flipped learning in English as a foreign language classroom: outcomes and perceptions. *TESOL Quarterly* 52 62–84. 10.1002/tesq.372

[B40] Lin H. C. Hwang G. J. Hsu Y. D. (2019). Effects of ASQ-based flipped learning on nurse practitioner learners’ nursing skills, learning achievement and learning perceptions. *Comp. Educ.* 139 207–221. 10.1016/j.compedu.2019.05.014

[B41] Mason G. S. Shuman T. R. Cook K. E. (2013). Comparing the effectiveness of an inverted classroom to a traditional classroom in an upper-division engineering course. *IEEE Trans. Educ.* 56 430–435. 10.1109/TE.2013.2249066

[B42] Mengual-Andrés S. López-Belmonte J. Fuentes-Cabrera A. Pozo-Sánchez S. (2020). Modelo estructural de factores extrínsecos influyentes en el flipped learning. *Educ. XX* 1 75–101. 10.5944/educxx1.23840

[B43] Milman N. B. (2012). The flipped classroom strategy: what is it and how can it best be used? *Distance Learn.* 9 85–87.

[B44] Namik K. Boae C. Jeong-Im C. (2014). A case study of flipped learning at college: focused on effects of motivation and self-efficacy. *Educ. Technol.* 30 467–492. 10.17232/KSET.30.3.467

[B45] O’Flaherty J. Philips C. (2015). The use of flipped classrooms in higher education: a scoping review. *Int. Higher Educ.* 25 85–95. 10.11124/JBISRIR-2017-003903 32813351

[B46] Ouda H. Ahmed K. (2016). Flipped learning as a new educational paradigm: an analytical critical study. *Eur. Sci. J.* 12 417–444. 10.19044/esj.2016.v12n10p417

[B47] Pérez D. P. Riveros R. M. (2014). “Unleashing the power of blended learning and flipped classroom for english as a foreign language learning: three spheres of challenges and strategies,” in *Proceedings of the a Higher Education Institution in Colombia. Proceedings of ICERI2014 Conference 17th-19th November 2014*, (Seville).

[B48] Qader R. O. Arslan F. Y. (2019). The effect of flipped classroom instruction in writing: a case study with Iraqi EFL learners. *Teach. English Technol.* 19 36–55.

[B49] Roach T. (2014). Student perceptions toward flipped learning: new methods to increase interaction and active learning in economics. *Int. Rev. Econ. Educ.* 17 74–84. 10.1016/j.iree.2014.08.003

[B50] Schwanki E. R. (2013). *Blended Learning: Achievement and Perception Flipped Classroom: Effects on Achievement and Student Perception.* Master’sdissertation, Marshall, MN: Southwest Minnesota State University.

[B51] Segolsson M. Hirsh A. Backlund J. (2017). The flipped classroom and students learning at compulsory School in Sweden- a Longitudinal, qualitative study. *J. Educ. Practice* 8 77–86.

[B52] Shotaro A. Fumiya S. Haruya Y. (2018). Classroom approach on EFL japanese junior high school students’ performances and classroom approach on EFL Japanese Junior High School Students’ performances and attitudes. *Int. J. Heritage Art Multimedia* 1 71–87.

[B53] Tully D. (2016). *The Effects Of A Flipped Learning Model Utilizing Varied Technology Versus the Traditional Learning Model in a High School Biology Classroom.* Boseman, MT: Montana State University. Unpublished MA Thesis.

[B54] Vaezi R. Afghari A. Lotfi A. (2019). Flipped teaching: Iranian students’ and teachers’. *Perceptions* 8 139–164. 10.22108/are.2019.114131.1382

[B55] Valenza J. K. (2012). The flipping librarian. *Teacher Librarian* 40 22–25.

[B56] Wette R. (2011). Product–process distinctions in ELT curriculum theory and practice. *ELT J.* 65 136–144. 10.1093/elt/ccq022

[B57] Willey K. Gardner A. (2013). “Flipping your classroom without flipping out,” in *Proceedings of the 41st SEFI Conference, 16-20 September 2013, (16-20)*, (Leuven). 10.3205/zma001526

[B58] Wu W. C. Marek M. Yen L. L. (2012). Promotion of EFL student motivation, confidence, and satisfaction via a learning spiral, peer-scaffolding, and CMC. *Int. J. Computer-Assisted Lang. Learn. Teach. (IJCALLT)* 2 54–75. 10.4018/ijcallt.2012070104

[B59] Yu Z. Wang G. (2016). Academic achievements and satisfaction of the clicker-aided flipped business English writing class. *Educ. Technol. Soc.* 19 298–312.

